# The influence of normative and subjective oral health status on schoolchildren’s happiness

**DOI:** 10.1186/1472-6831-15-15

**Published:** 2015-01-23

**Authors:** Simone Tuchtenhagen, Carmela Rampazzo Bresolin, Fernanda Tomazoni, Guilherme Nascimento da Rosa, Joana Possamai Del Fabro, Fausto Medeiros Mendes, José Leopoldo Ferreira Antunes, Thiago Machado Ardenghi

**Affiliations:** Department of Stomatology, Federal University of Santa Maria, UFSM, Rua Cel.Niederauer 917/208, Santa Maria, RS Brazil; Department of Pediatric Dentistry, School of Dentistry, University of São Paulo, São Paulo, SP Brazil; Department of Epidemiology, School of Public Health, University of São Paulo, São Paulo, SP Brazil

**Keywords:** Happiness, Child, Oral health, Quality of life

## Abstract

**Background:**

Traditional methods to measure oral health based on clinical standards are limited because they do not consider psychosocial and functional aspects of oral health. It has been recommended that these measures need to be supplemented by data obtained from patients regarding their individual perceptions on oral health-related quality of life (OHRQoL). Happiness is a multidimensional construct comprising both emotional and cognitive domains, and has been defined as “the degree to which an individual judges the overall quality of his or her life as a whole favorably”. It has been associated with several health outcomes, including oral health. The aim of this study was to assess the impact of oral health conditions, oral health-related quality of life (OHRQoL), and socioeconomic factors on the subjective happiness of Brazilian adolescents.

**Methods:**

A cross-sectional study was conducted in 2012 on a representative sample of 12-year-old schoolchildren in Santa Maria-RS, Brazil. The data were collected through dental examinations and structured interviews. The participants underwent an evaluation aimed at detecting dental caries, traumatic dental injuries, malocclusion, and gingival bleeding. They also completed the Brazilian versions of the Child Perceptions Questionnaire-short form (CPQ11–14 – ISF: 16) and the Subjective Happiness Scale (SHS), which was our outcome variable. Socioeconomic conditions were evaluated through a questionnaire that was completed by the participants’ parents. Poisson regression analysis was used to determine the association between the explanatory variables and the outcome. Moreover, a correlation analysis was performed to determine the relationship between the SHS scores and the overall and domain scores of the CPQ11–14 –ISF: 16.

**Results:**

A total of 1,134 children were evaluated. Unadjusted analyses showed that happiness was associated with socioeconomic indicators, the use of dental services, clinical status, and scores on the OHRQoL measure. After adjustment, household overcrowding (RR: 0.96; 95% CI: 0.93-0.98), dental caries (RR: 0.98; 95% CI: 0.97-0.99), malocclusion (RR: 0.98; 95% CI: 0.96-0.99), and the severity associated with the CPQ11-14 (RR: 0.95; 95% CI: 0.93-0.97) still showed a significant association with lower levels of the mean SHS score.

**Conclusions:**

Happiness is influenced by oral conditions, socioeconomic status, and OHRQoL.

## Background

The traditional methods used to measure oral health on the basis of clinical standards are limited, as they do not consider the psychosocial and functional aspects of oral health. It has been recommended that these measures be supplemented by data obtained from patients, comprising their subjective perceptions regarding oral health-related quality of life (OHRQoL) [1–3]. Previous findings suggest that poor oral health among children affects their quality of life in a multidimensional way [4–6]. This means that it is not only the domains related to functional limitations that are compromised, but also those associated with their social and emotional well-being. In fact, research has shown that psychosocial characteristics are important contributors towards OHRQoL among adolescents and they appear to be more important than sociodemographic or clinical characteristics [7].

Happiness is a multidimensional construct comprising both emotional and cognitive domains, and has been defined as “the degree to which an individual judges the overall quality of his or her life favorably, as a whole” [8]. Happiness has been associated with several health outcomes, such as regular exercise, not smoking, reduced alcohol intake, higher sleep quality and quantity, and a healthy diet [9–13]. Generally, subjective measures of health, such as self-reported health or health-related quality of life, are closely related to happiness [14–16].

Oral health outcomes have the potential to influence happiness. Yoon et al. [16] found a significant relationship between oral health-related factors and happiness among an elderly Korean sample, even when the analyses were adjusted by demographic, socioeconomic, and general health-related variables. One of the most important findings of that study was that approximately 10% of the variation in happiness was explained by the participants’ oral health status and oral health behaviors [16]. Conversely, Honkala et al. [17] showed that happiness is an important predictor of oral health behaviors. Using data from adolescents aged 11 to 13 years old, the authors found that “feeling very happy” was a predictor of the participants’ inclination towards brushing their teeth on a regular basis.

To the best of our knowledge, there are no studies evaluating the influence of oral health conditions on the happiness of schoolchildren. Such a study is important, since happiness could be considered a satisfactory outcome of health interventions and policies. The identification of factors that contribute towards children’s happiness could facilitate an understanding of differences in children’s happiness levels, as well as help identify children who could benefit from interventions [18]. Therefore, the aim of this study is to assess the impact of oral health conditions, OHRQoL, and socioeconomic factors on the subjective happiness of 12-year-old Brazilian adolescents.

## Methods

### Sample selection and ethical considerations

A cross-sectional study was conducted on a representative sample of 12-year-old schoolchildren from Santa Maria, a southern city in Brazil, in 2012. The city has an estimated population of 261,031, including 3,817 children in the age range similar to that of the study sample [19]. For the calculation of the sample size when analyzing the effect of oral health conditions on happiness, the following parameters were used [20]: the average of 18.3 (SD = 4.7) obtained by the unexposed group (those who reported perceiving their dental health as good) on the Subjective Happiness Scale (SHS), the average of 17.0 (SD = 4.8) and a 95% confidence interval (95% CI) obtained by the exposed group (those who reported perceiving their dental health as poor/very poor), a ratio of 1:1 between the exposed and unexposed group, a standard error of 5%, and a design effect of 1.2, with 30% added to possible declines. The minimum sample size required was 656 children.

A two-stage sampling procedure was adopted. All the public schools in the city constituted the sampling units used in the first stage of sampling. Twenty schools were randomly selected for participation in the study [21]. Because the schools’ varying sizes, an equal probability selection method (i.e., probability proportional to size) was used to ensure that each school had an equal chance of being selected. The second-stage sampling units comprised all the 12-year-old children enrolled at each of the selected schools.

The study protocol was reviewed and approved by the Research Ethics Committee at the Federal University of Santa Maria. All children gave consent to participate in the study. Moreover, each of their parents or guardians signed an informed consent form.

### Data collection

Data were collected from dental examinations and structured interviews. The children underwent the dental examinations at their schools; these were conducted by four trained examiners who were trained and calibrated for data collection before the survey. The dental examinations were performed in a room with natural light, using periodontal probes and dental mirrors. These examinations included assessments of dental caries, dental trauma, malocclusion, and gingival conditions, in accordance with the international criteria for oral health surveys, as standardized by the World Health Organization (WHO) [21]. The training and calibration process lasted 36 hours and included theoretical activities, discussions regarding diagnostic criteria for all of the conditions, and the examination of 20 children. A benchmark dental examiner carried out the process.

The prevalence of untreated dental caries (corresponding to a non-zero D component in the DMFT) was recorded in accordance with the WHO criteria. Traumatic dental injuries were assessed through the O’Brien index and recorded as “present”, which represents any type of fracture, or “absent” [22]. Malocclusion was assessed through the Dental Aesthetic Index (DAI) and the children were recorded as having malocclusion if the final DAI score was higher than 25, thus indicating a need for elective, highly desirable, or mandatory orthodontic treatment [21]. Children with 15% or more gingival bleeding sites upon probing were categorized as having gingivitis [23].

Socioeconomic characteristics were provided by parents and guardians. The questionnaire provided information on gender, race, parents’ educational level, household income, household overcrowding, and consultations with a dentist. Race was recorded according to the criteria used by the Brazilian Institute of Geography and Statistics (white, black, mixed, or other) [19], and the participants were categorized as “white” or “non–white”. Details regarding educational levels enabled the comparison of fathers and mothers who had completed 8 years of formal instruction, which refers to primary school education in the Brazilian context, with those who had not. Household income was measured in terms of Brazil’s minimum wage, which is standard practice for this type of assessment. The minimum wage amounted to nearly US$ 450 during the data collection period. The threshold for household income was obtained by the median (1.6 BMW). Household overcrowding was determined through the number of rooms per person in a household. Children who had visited the dentist within the last 6 months were compared with those who had not. The feasibility of the socioeconomic questionnaire had previously been assessed through use on a sample of 20 parents during the calibration process. These parents and children were not included in the final sample.

Subjective happiness was assessed through the Brazilian version of the Subjective Happiness Scale (SHS) [24, 25], which consists of four items rated on a 7-point Likert scale requiring individuals to indicate whether they agreed or disagreed with the statements. The scale consisted of the following items: “In general, I consider myself a very happy person”, “Compared to most of my peers, I consider myself happier”, “Some people are generally very happy. They enjoy life regardless of what is going on, getting the most out of everything. To what extent does this account describe you?” and “Some people are generally not very happy. Although they are not depressed, they never seem as happy as they might be. To what extent does this characterization describe you?” This last question is reverse coded, as proposed previously [12, 24]. An overall SHS score is computed by taking the mean of responses to the four items; scores can range from 1 to 7, with higher values corresponding to better subjective happiness [24].

Children also completed the short form the Brazilian version of the Child Perceptions Questionnaire 11–14 (CPQ11–14 – ISF:16) [26, 27]. The short version of the CPQ11–14 – ISF:16 comprises 16 questions that can be classified into four domains: oral symptoms (4 questions), functional limitation (4 questions), emotional well-being (4 questions) and social well-being (4 questions). Each question had five possible answers, each with a score ranging from 0 to 4; a higher score indicated a poorer status. Scores on the CPQ11–14 – ISF: 16 are determined through the sum of scores obtained for each domain. The overall score on the CPQ11–14 – ISF: 16 ranges from 0 to 80; higher scores denote a greater impact of oral conditions on children’s quality of life.

The children responded to the CPQ11–14 – ISF: 16 questionnaire and the SHS during face-to-face interviews conducted by the examiners, and cue cards listing possible responses were used to guide the participants. If the children answered “don’t know” to any question, it was coded as missing.

### Data analysis

The data were analyzed with Stata 12 (Stata Corporation; College Station, TX, USA). Descriptive analysis provided statistics indicating the sample demographics, as well as clinical and socioeconomic characteristics. Furthermore, the mean SHS scores and the overall CPQ11–14 – ISF: 16 and domain scores were estimated. Unadjusted analyses were conducted so as to provide summary statistics and preliminary assessment of the association between predictor variables and the outcome (mean SHS score). Correlation analysis was performed to measure the degree of correlation among the mean SHS and overall CPQ11–14 – ISF: 16 score and specific domains. Models were fitted by Poisson regression analysis. This analytical approach allowed estimating the rate ratio (RR) and respective 95% CIs to assess the explanatory variables that are associated with happiness. It corresponds to the ratio of the arithmetic mean of SHS scores between exposed/unexposed group. All analyses took into account the sample weight, using the “svy” commands in Stata for complex data samples.

## Results

A total of 1,134 children, 45.88% boys and 54.12% girls, were evaluated. A response rate of 93.00% was attained. Non-participation was mainly due to the absence of some children on the day scheduled for the examination, or those who forgot to bring the consent form signed by their parents. The response ratio was similar for each school considered in the sample.

The children were predominately white (77.93%), with almost half living in a household with an income 1.6 times higher than Brazil’s minimum wage (BMW). Nearly 69.05% of the children lived in a house with one or more rooms per person. The prevalence of untreated dental caries (component “D” of the DMF index), traumatic dental injuries, malocclusion, and severity of gingivitis (≥15% of the sites bleeding) were 42.28, 25.16, 42.36, and 26.24% respectively. The overall DMF-T was 1.15 (95% CI: 1,01-1.29). Scores on the CPQ11–14 – ISF: 16 ranged from 0 to 43, with an average of 10.23 (standard error = 0.32). These data are summarized in Table 1.

**Table 1:** **Sociodemographic, clinical and subjective characteristics of the sample: 1134 12-years-old children, Santa Maria – RS, Brazil**

Variables	n	%*
**Gender**		
Female	611	54.12
Male	523	45.88
**Skin color**		
White	851	77.93
Non-white	254	22.07
**Household income**		
>1.6 BMW**	487	47.78
≤1.6 BMW**	549	52.22
**Mother’s schooling**		
≥8 years	702	65.55
<8 years	382	34.45
**Father’s schooling**		
≥8 years	628	61.44
<8 years	406	38.56
**Household overcrowding**		
1 room or more/person	736	69.05
Less than 1 room/person	337	30.95
**Visited a dentist in the last 6 months**		
Yes	514	47.43
No	574	52.57
**Cavitated carious lesions**		
Without	654	57.72
With	480	42.28
**Traumatic dental injury**		
Without	848	74.84
With	286	25.16
**Malocclusion**		
Without	656	57.64
With	478	42.36
**Gingival bleeding**		
<15% sites	836	73.76
≥15% sites	298	26.24
**Variables**	**Mean**	SD***
**CPQ11–14 – ISF:16 (overall scale)**	10.23	7.68
Oral symptoms	3.48	2.50
Functional limitation	2.45	2.43
Emotional well-being	2.68	3.05
Social well-being	1.62	2.12
**Subjective happiness scale**	5.24	0.90

*Taking into account the sampling weight.

** BMW: Brazilian minimum wage (approximately U$ 450 during the data gathering).

***SD: Standard deviation.

The mean SHS score was 5.24 (95% CI: 5.14-5.33, standard deviation = 0.90); scores ranged from 1.75 to 7. Responses to all of the questions ranged from 1 (minimum) to 7 (maximum). The highest mean scores were obtained for Question 1 (“In general, I consider myself a very happy person”), while the lowest were obtained for the last question (“Some people are generally not very happy. Although…”) (Table 2).

**Table 2:** **Descriptive distribution (mean and standard deviation) of total SHS scores**

SHS question	Mean (sd)	Range
“In general, I consider myself a very happy person”	5.91 (1.22)	1-7
“Compared to most of my peers, I consider myself…”	5.45 (1.41)	1-7
“Some people are generally very happy. They enjoy…”	5.22 (1.55)	1-7
“Some people are generally not very happy. Although…”	4.36 (1.86)	1-7
**Total SHS**	5.24 (0.90)	1.75-7

SHS: Subjective Happiness Scale; sd: standard deviation.

Unadjusted Poisson analyses showed a significant association between happiness and socioeconomic indicators (skin color, household income, and household overcrowding), use of dental services (consulted a dentist within the last 6 months), clinical status (dental caries and malocclusion), and the CPQ11–14 – ISF:16 severity (percentage of children who answered “often” or “every day/almost every day” to any of the questions on the questionnaire) (Table 3).

**Table 3:** **Unadjusted and adjusted Poisson regression analyses of the association between clinical, socioeconomic and subjective measures and happiness**

Variables	Mean SHS score* (SE)	RRunadjusted (95% IC)	RRadjusted (95% IC)
**Gender**			
Female	5.26 (0.06)	1	
Male	5.21(0.05)	0.99 (0.96 – 1.02)	
**Skin color**			
White	5.27 (0.05)	1	
Non-white	5.11 (0.06)	0.97 (0.94 – 1.00)	
**Household income**			
>1.6 BMW**	5.15 (0.05)	1	
≤1.6 BMW**	5.35 (0.06)	0.96 (0.94 – 0.99)	
**Mother’s schooling**			
≥8 years	5.32 (0.04)	1	
<8 years	5.11 (0.06)	0.96 (0.93 – 0.99)	
**Father’s schooling**			
≥8 years	5.30 (0.06)	1	
<8 years	5.16 (0.06)	0.97 (0.94 – 1.01)	
**Household overcrownding**			
1 room or more/person	5.32 (0.04)	1	1
Less than 1 room/person	5.06 (0.06)	0.95 (0.93 – 0.97)	0.96 (0.93 – 0.98)
**Visited a dentist in the last 6 months**			
Yes	5.31 (0.04)	1	
No	5.17 (0.05)	0.97 (0.96 – 0.99)	
**Cavittaed carious lesions**			
Without	5.29 (0.05)	1	1
With	5.16 (0.04)	0.97 (0.96 – 0.99)	0.98 (0.97 – 0.99)
**Traumatic dental injury**			
Without	5.24 (0.05)	1	1
With	5.22 (0.06)	0.99 (0.97 – 1.02)	0.99 (0.97 – 1.01)
**Malocclusion**			
Without	5.29 (0.05)	1	1
With	5.16 (0.04)	0.97 (0.96 – 0.99)	0.98 (0.96 – 0.99)
**Gingival bleeding**			
<15% sites	5.26 (0.04)	1	1
≥15% sites	5.16 (0.07)	0.98 (0.95 – 1.00)	0.98 (0.96 – 1.01)
**CPQ11–14 – ISF:16**			
Never/once/twice/sometimes	5.38 (0.05)	1	1
Often/every day/almost every day	5.07 (0.04)	0.94 (0.93 – 0.96)	0.95 (0.93 – 0.97)

*Taking into account the sampling weight.

**BMW: Brazilian minimum wage (approximately U$ 450 during the data gathering).

Following adjustment, there remained an association between household overcrowding and the outcome, with children in households with less than one room per person obtaining lower mean scores on the SHS. Moreover, lower levels of happiness could be attributed to children with dental caries and malocclusion. The severity of the score obtained on the CPQ11–14 – ISF:16 was significantly associated with lower means on the SHS (Table 3).

Table 4 presents Pearson’s correlation coefficient for the mean SHS score and overall scores on the CPQ11–14 – ISF:16 and domain scores. A significant negative correlation was found between the mean SHS score and overall CPQ11–14 – ISF:16 (*r* = -0.29; *p* = 0.000). This meant that a decrease in the mean overall CPQ11–14 – ISF:16 score was associated with an increase in the mean SHS score (i.e., better OHRQoL leads to better self-reported happiness).

**Table 4:** **Pairwise Pearson correlation among results obtained with subjective happiness scale and overall and domains of CPQ 11–14**

	CPQ11–14 – ISF:16	SHS	OS	SWB	EWB	FL
**CPQ11–14 – ISF:16**	1.00					
**Score SHS**	-0.29*	1.00				
**OS**	0.72*	-0.21*	1.00			
**SWB**	0.73*	-0.23*	0.35*	1.00		
**EWB**	0.81*	-0.27*	0.38*	0.56*	1.00	
**FL**	0.72*	-0.15*	0.43*	0.35*	0.40*	1.00

CPQ11–14 – ISF: 16: Child Perceptions Questionnaire; SHS: Subjective Happines Scale; OS: Oral symptoms; SWB: Social well-being; EWB: Emotional well-being; FL: Funcional limitation.

*p < 0.001.

## Discussion

This cross-sectional study assessed the impact of oral health status and socioeconomic profiles on happiness among Brazilian adolescents. Our primary findings showed that happiness is influenced by oral conditions, socioeconomic status, and OHRQoL. To the best of our knowledge, this is the first study assessing these associations among children and adolescents.

The presence of untreated dental caries was associated with lower levels of happiness. Several studies have reported the negative impact of poor dental status on quality of life. This condition is also associated with higher overall and domain-specific CPQ11–14 – ISF: 16 scores. Children with cavitated carious lesions are more likely to experience dental pain and chewing difficulties. They are also more likely to have been worried or upset about their oral health status, which further impairs their quality of life [28, 29]. Studies have also reported that cavitated caries lesions can also affect certain emotional aspects among adolescents, which in turn could explain the former’s influence on happiness [27, 29].

An association was found between malocclusion and happiness. For aesthetic reasons, malocclusion may play an important role in social interaction and acceptance, with the potential to result in functional limitations in more severe cases [30–34]. Scapini et al. [34] found that the association between malocclusion and the CPQ11–14 – ISF: 16 scores was significant mainly due to the social and emotional well-being domains. Studies affirm that a disturbance of normal occlusion may reduce social acceptance and induce low self-esteem and poor quality of life through psychosocial pathways [28, 34, 35]. This could be due to individuals’ poor perceived attractiveness. In their study, Holder and Coleman [18] demonstrated an association between attractiveness and happiness, with children who reported perceiving themselves as good-looking proving to be happier than their counterparts.

There was a significant correlation between the mean SHS score and the overall CPQ11–14 – ISF: 16 score. A similar correlation was found by Yoon et al. [16], with the OHRQoL measure identified as one of the major explanatory variables of happiness in their final model, since the only oral health related measure that remained significant in the model were the scores from the OHRQoL questionnaire. The authors conclude that subjective health indicators may be better predictors of happiness, as compared to objective ones. This suggests that the way people perceive their oral health is an important indicator of subjective happiness; those who perceive their oral health as poor, feeling less happy than those who perceive their oral health as good/very good. This highlights the role of self-perceived oral health as an important feature of children’s welfare. Therefore, oral health could have a broader impact on individuals’ lives, not only influencing domains related to OHRQoL, but also overall well-being. In our study, we noted an association between happiness and both objective (presence of dental caries and malocclusion) and subjective (OHRQoL) factors, as these variables remained statistically significant in the adjusted regression model.

We also found that socioeconomic conditions have an impact on a child’s happiness. In the unadjusted model, income was negatively associated with happiness; children living with a household income ≤ 1.6 BMW reported lower mean scores on the SHS when compared to their counterparts. Although there is no consensus on the threshold used for classifying individuals as poor or rich according to the income, previous studies have reported that income inequality, relative poverty, and social comparison have an impact on the individuals’ psychological well-being [29, 36–38]. Socioeconomic status has been linked with health outcomes (as happiness) by different pathways. For instance, there is conflicting results of the effect of income on health status. The materialist theory states that “health inequalities result from the differential accumulation of exposures and experiences that have their sources in the material world” [39]. Therefore, for each increase in the income would lead to an increase in the considered health outcome. The psychosocial explanation states that social inequality may affect how people feel in comparison with their social strata, which in turn can affect health through stress induced behaviour and neuroendocrine pathways [40, 41].

In the final model, household overcrowding was negatively associated with happiness; children in a household with less than one room per person reported lower mean scores on the SHS. A theoretical explanation of the link between socioeconomic status and oral health outcomes focuses on the effect of psychosocial variables on individual lifestyle decisions [41]. That is, people who live in deprivation tend to make unhealthy choices and perceive their health as poorer compared to their counterparts. This observation could also be applicable to subjective happiness.

Some aspects of the methodology of this investigation merit discussion; thus, the results must be interpreted with caution. We have considered only public schools in the sample. We have tried to obtain a representative sample using a random sample of all public schools of the city, and the selected schools were distributed in all administratively regions of the city. Although we did not selected private schools, approximately 85% of the schoolchildren in the city attend public schools. Nevertheless, we have compared our sample with the data of provided by Demographic Council of the City in terms of race, sex, and household income: the sampled subjects did not differ of the city’s population according to these characteristics (Chi-squared test). Therefore, a selection bias is unlikely to be occurred. In this study, we used a cross-sectional design, which preempts assumptions relating to causality and temporal relations between outcome and predictor variables. Therefore, one may argue that the nature of the relationship between OHRQoL and happiness could be inverse. Nevertheless, it is widely known that oral conditions affect the manner in which children perceive their OHRQoL. Since oral health conditions can have an impact on happiness, we believe that better OHRQoL is likely to lead to higher levels of happiness. We used a measure of happiness that was not validated to this specific age group yet, although the Brazilian validation study [25] included a sample with a age range of 15–66 years. Besides, studies from other languages have addressed happiness in adolescents from different age groups with the SHS [42–46], and the results support that the SHS has good psychometric properties, high internal consistency, stability over time and across samples.

## Conclusions

This study showed an association between happiness and oral health conditions, socioeconomic status, and ORHQoL. We believe that these findings are particularly important for use in health planning. Happiness may be considered a satisfactory outcome regarding health interventions and policies, as well as a goal to be attained through implementation of interventions. This is particularly the case when considering that clinical measurements of oral disease do not fully take into account children’s oral health needs. The identification of conditions affecting individuals’ happiness facilitates opportunities to plan policies aimed at benefitting a large number of children and, ultimately, contribute towards the effectiveness of public health programs.

## Authors’ information

Simone Tuchtenhagen, DDS, MSc Student, Department of Stomatology, Federal University of Santa Maria, UFSM, Santa Maria, Brazil, and Department of Epidemiology, School of Public Health, University of São Paulo, SP, Brazil. Carmela Rampazzo Bresolin, DDS, MSc Student, Department of Stomatology, Federal University of Santa Maria, UFSM, Santa Maria, Brazil, and Department of Pediatric Dentistry, University of São Paulo, São Paulo, Brazil. . Fernanda Tomazoni, DDS, MSc Student, Department of Stomatology, Federal University of Santa Maria, UFSM, Santa Maria, Brazil. Guilherme Nascimento da Rosa, DDS, MSc Student, Department of Stomatology, Federal University of Santa Maria, UFSM, Santa Maria, Brazil. Joana Possamai Del Fabro, DDS, MSc Student, Department of Stomatology, Federal University of Santa Maria, UFSM, Santa Maria, Brazil. Fausto Medeiros Mendes, DDS, PhD, Associate Professor, Department of Pediatric Dentistry, University of São Paulo, São Paulo, Brazil. José Leopoldo Ferreira Antunes, PhD, Full Professor, Department of Epidemiology, School of Public Health, University of São Paulo, SP, Brazil. Thiago Machado Ardenghi, DDS, PhD, Adjunct Professor, Department of Stomatology, Federal University of Santa Maria, UFSM, Santa Maria, Brazil.

## Abbreviations

OHRQoL: Oral Health-Related Quality of Life
SHS: Subjective Happiness Scale
CPQ11–14 – ISF: 16: Child Perdeptions Questionnaire 11–14, short form
RR: Rate ratio
CI: Confidence Interval
SD: Standard Deviation
WHO: World Health Organization
DMFT: Decayed, Missing and Filled Teeth Index
DAI: Dental Aesthetic Index
BMW: Brazilian Minimum Wage.
